# Teachers’ Perspectives on the Impact of an Outdoor-Based Self-Care Training Program on Student Mental Health

**DOI:** 10.3390/ijerph22071135

**Published:** 2025-07-18

**Authors:** Katie A. Bucher, Connor M. Moriarty, Adam Lazarchak, Russell K. McIntire

**Affiliations:** 1 Muhlenberg College, Allentown, PA 18104, USA; 2 Reset Outdoors, Bethlehem, PA 18015, USA; connor@resetoutdoors.com; 3 Bethlehem Area Vocational Technical School, Bethlehem, PA 18020, USA; lazarchaka@bavts.org; 4 College of Health, Lehigh University, Bethlehem, PA 18015, USA; rum222@lehigh.edu

**Keywords:** mental health, self-care, well-being, nature, adolescents, teachers

## Abstract

As mental health disorders in the U.S. increase at an alarming rate, schools are looking for prevention and mitigation interventions for their students. This study reports teachers’ perspectives on the effectiveness of a school program situated at the intersection of self-care and nature-based interventions at a vocational technical high school. Eight classes received between one and four “Intro to Well-Being” sessions, designed to take students outside and develop self-awareness, sense of connection, and reduce stress. Upon the conclusion of the program, classroom teachers were interviewed to identify the perceptions of the sessions and their impact on student and classroom outcomes. Teachers described the sessions as beneficial to students’ expression of emotions, mindfulness skills, personal/social connections, and classroom atmosphere. Teachers also reflected on challenges and provided recommendations for future implementation. Outdoor-based self-care programs, administered at schools, may be a promising program model to support the social and emotional health of students.

## 1. Introduction

Since 2009, the percentage of U.S. teenagers who reported major depressive episodes in the past year has been increasing at an alarming rate [1]. In its Youth Risk Behavior Survey-Data Summary and Trends Report 2009–2019, the CDC reported that mental health variables for high school students were trending “in the wrong direction”. The report stated that the percentage of high school students who experienced “persistent feelings of sadness or hopelessness” had increased from 26.1% in 2009 to 36.7% in 2019 across all races/ethnicities [2]. As Duong et al. point out, mental health disorders among children and adolescents are at a historical high and continue to increase [3].

Such alarming trends are reflected in recent school-reported statistics as well. In 2022, the U.S. Department of Education noted dramatic increases in mental health issues in primary and secondary schools after students returned to in-person instruction during the 2021–2022 school year; more than two-thirds of public schools reported an increase in the percentage of students seeking mental health services from school since the start of the pandemic [4]. Deepening the concern, just over half of public schools felt that their school could effectively provide mental health services to all students who needed them [4]. Among the most common reasons given for limited ability to effectively provide needed services was insufficient mental health staffing and inadequate funding [4]. Clearly, the rising mental health crisis at schools plays out every day in classrooms across the country.

Given the trends of increased mental health concerns parallel to limited resources to meet rising needs, schools and community organizations have been increasingly implementing adolescent mental health interventions that do not rely on mental health treatment [5,6]. Some of these interventions focus on teaching self-care, which, at the individual level, has been defined as “caring for self when ill or positive actions and adopting behaviors to prevent illness” [7]. For example, programs to encourage self-care among adolescents to exercise, meditate, journal, or get adequate sleep could reduce stress, prevent mental health conditions, or help those with conditions manage symptoms [5,8]. While self-care intervention programs have much potential to address the rising need for mental health support for adolescents, more research is necessary to guide school personnel about the effectiveness of these programs.

Another type of intervention that shows potential to mitigate mental health concerns among youth is nature- or outdoor-based programs. A strong research base shows that time in nature and nature experiences are important for the mental health of children and adolescents [9,10,11]. In recent years, schools have begun providing increased opportunities for student nature experiences both during and after school [12,13]. These programs encourage students to get outside and interact with nature. Studies show positive impacts of these programs on children’s educational achievement, intrinsic motivation, and school attendance, highlighting the value of such programs for student engagement and academic achievement generally [10,14,15,16,17]. However, less research has been conducted on the mental health benefits of outdoor-based programs [18]. Studies also suggest that teachers’ perspectives on the implementation of school-based mental health programs [19] and outdoor education [20], respectively, are underexamined and particularly important to understand the effectiveness of those programs on student learning and well-being.

This study aimed to provide insight into the effectiveness of a pilot nature-based self-care intervention at a vocational technical high school. This is the first empirical evaluation of this pilot program. The self-reported health of participating students is being investigated in a parallel study; the current study focused on the perspectives of participating teachers regarding the impacts of the intervention on students individually and collectively as a class. By highlighting teachers’ perspectives, we aimed to provide more nuanced insights into the processes and experiences of program implementation, i.e., insights that may be helpful to personnel at other schools looking for viable ways to address mental health concerns among their student body.

## 2. Materials and Methods

For the 2023–2024 school year, a vocational technical high school (hereafter referred to as VTHS) in a mid-sized Pennsylvanian city collaborated with the Reset Outdoors organization to pilot mental health and wellness programs with eight high school classes. Reset Outdoors is a clinical mental health practice and an organizational development firm [21]. Clinically, they provide indoor, tele-health, and outdoor-based mental health services that promote well-being among clients and organizations, including schools.

During winter/spring 2024, eight classes at VTHS received between one and four “Intro to Well-Being” sessions designed by Reset Outdoors facilitators to develop students’ self-awareness, sense of belonging and connection, and reduce stress. The total number of Reset Outdoors sessions at VTHS was determined by staffing considerations. The number of sessions each class received was determined by school scheduling feasibility; in this study 3 classes received 1 session, 2 classes received 2 sessions, and 3 classes received 4 sessions. The classes that received the sessions were from a range of vocational technical classes, including culinary arts, building trades, health careers, electronics technology, and protective services. Classes were selected based on teachers’ voluntary interest in hosting Reset Outdoors sessions in their classes.

Students began each session by taking a baseline well-being self-assessment using an instrument developed by Reset Outdoors. Facilitators then prepped students for a 20–40 min outdoor walk by providing guidance on mindful focus techniques intended to enhance the therapeutic value of the walk. These core techniques included a simple body scan activity which asked students to focus their attention on physical sensations in regions of their bodies (and also, the absence of sensations). Second, facilitators used an “8 Senses Check” to discuss the 8 bodily senses (sight, hearing, smell, taste, touch, interoception, exteroception, and proprioception) that students could use to identify information about interactions between their bodies and the environment. Facilitators encouraged the first lap of the subsequent walk (approximately 7–10 min) to be conducted silently as a way to increase students’ awareness of their surroundings and personal sensory experiences. Students’ reflections about the outdoor walk experience often focused on the core techniques described above, and provided the foundation for further classroom discussion about self-care strategies to reduce stress and improve well-being. These strategies were often student-driven, emerging naturally from the reflection conversations. Some examples of self-care strategies included asking for help from a classmate, teacher or friend, listening to music to clear the mind, taking a walk, or socializing with friends to generate social connections and feel belonging. Finally, facilitators repeated the well-being self-assessment for a second time, allowing each participant to identify how they felt at the conclusion of the session and compare those results with the assessment taken before the walk. Students were encouraged, but not required, to share the results of their self-assessments with others in the class. For classes that received more than one Well-Being session, facilitators followed a similar schedule but built upon discussions and skills developed during previous sessions.

**Figure 1:**
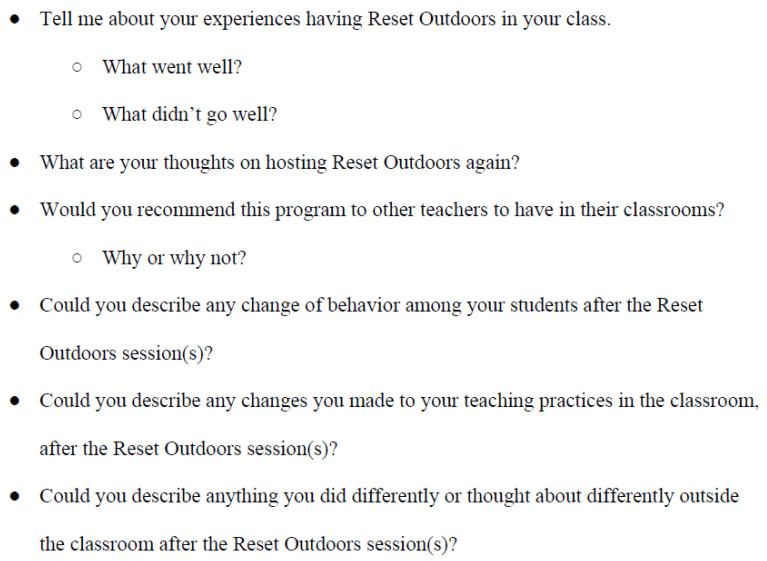
Semi-structured interview guide, May/June, 2024.

All classroom teachers were present for the Reset Outdoors sessions, participating in the walk and observing the classroom discussions. Subsequently, teachers had an opportunity to closely observe the pedagogical and therapeutic aspects of the sessions, as well as the reaction and responses of their students. Additionally, teachers could anecdotally observe any impacts—both momentary and lasting—of the sessions on their students. This study focused on elucidating teachers’ observations and experiences with the implementation of this program, and is based on interviews that a member of the research team conducted with each of the teachers who hosted Reset Outdoors sessions. The interviews used a semi-structured format to ask teachers about their perceptions of the sessions, specifically their observations of the impact of the sessions on students, individually and collectively as a class (Figure 1). Teachers were also asked about any challenges they observed and any recommendations for future programs. Teachers were interviewed, via Zoom, in late May and early June 2024 for a 15–30 min recorded interview. Interviews occurred between one week and three months after each teacher concluded class participation in the Reset Outdoors sessions. Interviews were transcribed and coded for patterned themes using the process of inductive analysis. Connor Moriarty is the Director of the Reset Outdoors Organization and was a primary facilitator during the sessions; for this reason, interviews were conducted and analyzed by other members of the research team. This study was conducted under a Lehigh University Institutional Review Board-approved protocol (2187202-1).

## 3. Results

### 3.1. Benefits

The primary benefit of the Reset Outdoors program discussed by interviewed teachers was the opportunity it provided for students to emotionally express themselves and “open up”. All interviewed teachers mentioned this benefit in some way, describing emotional expression as a key part of students’ mental health and their emotional “coping skills” more generally. All teachers emphasized such coping skills as an area of critical need for students they worked with, either as an important life skill in general or because individual students were experiencing particular mental and emotional stress. One teacher summarized the sentiments of many by explaining, “I really appreciated this opportunity to have Reset Outdoor come in, because to me the therapeutic component really really is something that is needed in my estimation as to what our students have to deal with.” For all participating teachers, the engagement of Reset Outdoors with their students constituted a valuable opportunity for classroom time to be focused on holistic support for their students. For example, one teacher summarized her experiences with the program the following way: “It was a really good introduction to the students about thinking about mental health and thinking about coping and thinking about feelings… it showed the importance of dealing with the mental health stuff by having a demonstrated strategy to deal with it.” Similarly, another teacher noted that the program provided students with “a platform to talk about them(selves) and their needs and how they can center themselves and focus on, you know, the ups and downs of life.” Several teachers noticed that students used skills learned through the Reset Outdoors sessions at other times later in the school year; one teacher said that after the sessions started, she would receive notes from students requesting time to destress outside or “circle to just talk” on days they were “struggling”.

Numerous teachers elaborated on the benefits of the outdoor silent walk, specifically. One teacher reported that this activity provided students with a more “natural way” to “take care of… some of their head stuff”, while another teacher explained that the walks encouraged students “to be themselves. But then, at the same time it wasn’t just a walk to walk, but it was also to say, ‘(I) want you to observe, I want you to listen. I want you to absorb what’s going on around you.’” Other teachers elaborated further on the benefits of sensory observation of focus in the walks, a skill several interviewed teachers describe as critical for the career fields their students are preparing to enter. For example, a teacher who teaches sensory assessment to her pre-nursing students described how the students were “amazed” at how the walk primed them for the type of detailed observation they practiced in the classroom: “They would talk to me and be like, Oh, my goodness, you’re so right… I can’t believe how I noticed the clouds, and how many different shapes were in the clouds… they noticed the feel underneath their feet of different places on our campus, and how different it was… They were amazed by the 15 min quiet walk.” Several other teachers mentioned that close observation during the walks also provided students with positive and unfamiliar experiences in nature, for example, “Some of the stuff that they talked about were the birds chirping. And it amazed me… but they don’t really have a reference for that. I feel like it… opened their eyes to what they don’t know outside. I liked that.” Similarly, another teacher noted that her students are “very rarely outside. So, they go take a walk outside, it’s totally weird to them. But they totally enjoyed it… they were like, ‘Wow! When can we do this again?’”

Teachers reported that program sessions also improved the overall energy level and atmosphere of their classrooms, particularly after students returned after the outdoor silent walk. One teacher observed that the walks “woke them up… they were awake to actually go accomplish what I set out for them”, while another teacher observed that after returning from the walks, he and the students “definitely had more energy”, and students were both “chattier” and “mellowed out.” Similarly, another teacher mentioned the following:

When we went on that walk and went for 3 laps and we came back in the classroom there was a totally different vibe, like people wanted to start participating… it definitely showed that just going out and taking that walk has very significant impact for something that only took a couple of minutes to do.

Other teachers emphasized that they observed improvements in students’ motivation and attitude toward school after participating in the Reset Outdoors sessions, a change they viewed as worth the time investment. For example, one teacher explained that while other teachers might think that going for a walk during school hours is “nonsense”, he viewed it as a way to make someone “a better student the other hours.”

Teachers emphasized that the program sessions also provided an opportunity for them to get to know students at a more individual level and for students to get to know each other, in part because program facilitators focused on honest communication. For example, one teacher mentioned that students “got to talk about something that they were like, ‘nobody’s ever asked me to talk about that before,” and explained that because students “started actually opening up”, she learned a lot more about their personal and out-of-classroom interests. Other teachers observed increased bonding between students, in part through the experience of hearing that other students had “problems” or personal challenges similar to their own. One teacher noted, “I think kids think they’re the only kid sitting in the classroom with a problem”, and explained that the Reset Outdoors sessions showed students that “you’re not alone.” Another teacher reported the following:

It was enlightening for the students to see that they all had a lot of things in common that they didn’t know before. That they enjoyed the same things. The group went outside, and they either stuck together because they didn’t like it, or they did like it, or they learned to relate to people. To each other.

Such personal bonding resulted in changes in how students interacted during other academic activities. For example, one teacher elaborated on the difference in student engagement and collaboration that she observed after the Reset Outdoors session with her class:

I had a really hard time up until they (Reset Outdoors) came in trying to get them (students) to actually work together, talk together. They just would stay in their bubble, and I’d have a few that would reach out and maybe do a little talking. But I have to say, after they had their session that all changed, they actually started working together and talking together and actually conversing like a normal group of students… it was immediate. From the next day on they acted that way.

Given that many of the interviewed teachers already included social–emotional learning (SEL) opportunities in their classroom, the Reset Outdoors program provided a form of professional validation of work they already saw as important. For example, one teacher said the program reinforced what he already valued as an educator: “For… people who are already doing it, it’s just reinforcing, it’s backing them up and saying, ‘Hey, great job! Keep doing what you’re doing.’” Other teachers said that while they already valued SEL, the Reset Outdoors sessions showed them new pedagogical approaches to try in the classroom. For example, one teacher explained what she learned from the Reset sessions: “I definitely learned to not talk as much and listen more. That’s one big thing I learned is that sometimes I just need to stop talking and I just need to let them talk… whether it be school related, whatever it is.” As another example, several teachers shared plans to continue with the outdoor walks in their regular lesson plans, with one teacher enthusiastically reporting “From what I’ve seen this year in terms of just the students’ attitudes changing, I’m actually going to be doing this next year… my students are gonna have to walk every day now, a.m. and p.m. …I’m gonna… keep that going.”

In sum, all teachers were very positive about the benefits of the Reset Outdoors program and identified numerous benefits, especially those related to mental health, a benefit which, in turn, influenced students’ engagement in classroom activities and improved classroom atmosphere more generally.

### 3.2. Challenges and Recommendations

While participating teachers were eager to implement the Reset Outdoors program again and discussed at length its many benefits, they did mention certain challenges to implementation and, subsequently, had recommendations for future implementation.

Two teachers mentioned the challenges of initial lack of “buy-in” from hesitant students, describing that at first some students were reluctant to try something new, especially the silent walk outdoors. Another teacher mentioned cold weather as a deterrent to students’ enthusiasm for going outdoors, although that same teacher lauded an instance when two students bonded over sharing a sweatshirt when one of them forgot their jacket. Several teachers mentioned that to address potentially hesitant students and increase the benefits of the program, multiple sessions are preferable. For example, a teacher whose class only had one Reset Outdoors session noted, “if you’re gonna do it you gotta have a minimum of at least 3 times, maybe even 4 times.” Two teachers noted that more than one session was specifically needed for organizers to build “rapport” and “connection” with students, especially those who needed time to “warm up” to the activities. While several teachers mentioned student hesitation to initially participate in the sessions, only one teacher mentioned challenges associated with disruptive behavior. However, this teacher noted that while he initially tried to “keep the kids quiet,” session organizers urged him to “let them be where they’re going to be” and side conversations subsequently were not a concern.

Notably, all teachers interviewed expressed interest in having the program not only repeated, but expanded in their school. Several teachers mentioned that a challenge to wider implementation was lack of supportive interest from other teachers, including “dismissive” attitudes about mental health activities in school more generally. For example, one teacher noted, “we have a lot of teachers out there that, I’m sure, would say, ‘oh this is nonsense.’” One teacher described dismissive attitudes as gendered, noting that some older male educators had been taught to “stuff” their feelings: “We unfortunately still have some of that mentality that’s been hard to penetrate.” Three teachers noted a possible way to increase “buy-in” from more teachers would be to include Reset Outdoors programming in mandatory in-service professional development. “I would love to see it being done on an in-service day where we do have to make all the teachers walk in silence for 15 min… I don’t think that they’re really going to get it unless they’re somewhat immersed into it. And I think an in-service day would be ideal,” one teacher suggested. Two teachers suggested that past participant teachers be included as presenters in any future programs, as they would be able to personally attest to the benefits of implementation. The program should “start from the adults and then get to the kids, if you know what I mean. Because many adults don’t think it’s necessary or important. They don’t see the value in it. But I think if you teach the teachers, then they can understand and embrace it more,” one teacher suggested.

## 4. Discussion

In this study, we report the results of interviews among teachers who hosted outdoor-based well-being sessions in their classrooms at VTHS in the winter/spring of 2024. Teachers described that the sessions were beneficial to students’ expression of emotions, mindfulness skills, personal/social connections, classroom atmosphere, and that the sessions validated the social and emotional content that teachers provided in class. Further, teachers reflected on challenges and provided recommendations for future implementation.

Foremost, teachers emphasized their belief that the Reset Outdoors sessions benefited students’ emotional/mental health. Interviews showed that teachers observed improvements in students’ emotive expression as a coping mechanism after Reset Outdoors conducted sessions in their class. Specifically, teachers reported that students were more open to talking about their personal feelings and challenges, a key component of developing broader social and emotional competencies among children and youth [22]. Studies show that empathetic and supportive communication between teachers and students supports the mental health of students, and can provide a foundation to bridge students to connect with counselors or other personnel for further support [23].

One of the goals of the well-being sessions was to develop students’ skills to deal with stressful situations. Teachers mentioned that the sessions, in particular the outdoor walks, fostered students’ mindfulness skills so they can focus on their surroundings and notice the environment around them. Mindfulness skills are important as a buffer for stressful events, and are negatively associated with adolescent depression, anxiety, and stress [24,25]. Emotional health and the ability to regulate emotions has also been shown to be important in the pathway between adolescent mindfulness and internalizing mental disorders, such as anxiety and depression [25]. In their systematic review, Djernis et al. [26] describe the positive psychological effects of nature-based mindfulness and note that “nature-based mindfulness is moderately superior to mindfulness conducted in non-natural settings,” a finding echoed by the teachers in this study.

Beyond personal health benefits, teachers described how the sessions improved class relationships and classroom atmosphere more generally. Numerous teachers highlighted the outdoor walk as providing a catalyst for students to build connections through common experiences as well as increased engagement and positive energy in the classroom. The literature has identified similar findings. As Zhang et al. [27] note, for adolescents, green space exposure is associated with stress relief, improved mood, and better emotional well-being, improvements which can contribute to a positive classroom atmosphere. The fact that participating teachers felt that they were more able to meet their regular instructional goals after the sessions underscored their desire to see this program not only repeated, but expanded.

From interviews, it is clear that teachers saw many positive benefits from participating in the Reset Outdoors program among their students, including their mental health, engagement with peers, and classroom atmosphere. All teachers expressed desire for the program to continue, with recommendations for expanding implementation at VTHS in the future. One particular challenge identified by a teacher for broader implementation of Reset Outdoors sessions was the perspectives of their fellow teachers whose “dismissive attitudes” about the importance of emotional wellness were “hard to penetrate.” Nevertheless, they also suggested that education and experience might convince more teachers to support hosting Reset Outdoors in their classrooms.

Overall, this study provides evidence that the Reset Outdoors pilot program was a positive experience impacting the classroom environment. However, our study has a number of limitations that should be noted. First, as previously mentioned, this study focuses on teachers’ perspectives only, and does not address researcher-observed or student-reported outcomes. Considering empirical outcomes from the student perspective, in addition to the current teacher-focused data, would more comprehensively evaluate the impact of the program and be informative for future iterations. Future studies should illuminate these impacts. Second, teacher participation in the program was voluntary. This population of early-adopter teachers likely supported the program at the onset, and therefore, more likely to have anticipated positive results and attributed them to the sessions, compared to non-participating teachers. This is a common limitation of voluntary pilot studies, and we caution that the small sample of teachers may not be representative of the full faculty at VTHS. Third, the time periods between the last Reset Outdoors session and the teacher interview varied per teacher, which may have affected the recall of the immediate impact of the sessions on teachers as reported in the interviews.

Feedback about the Reset Outdoors program has not been limited to the results of the interviews. Administrators at VTHS have received informal feedback from teachers, students, and staff about the program, and have decided to make changes to expand it. First, for the forthcoming academic years, the number of Reset Outdoors sessions administered to students will be doubled. This will increase the number of students exposed to the self-care curriculum, and the number of sessions per student. Second, for students who need a little extra support during the school day, Reset Outdoors will be contracted to provide one-on-one clinical support to students in collaboration with the school counseling department. Third, VTHS is considering a series of Reset Outdoors sessions for administrators, teachers, and/or staff members. The goal of the sessions would be to support and improve their mental health, but an added benefit could be the realization of the importance of self-care, and the acceptance of programs focused on improving social and emotional health of students.

The Reset Outdoors program is a potentially promising intervention model to foster self-care attitudes and behaviors among teens, teach them how to deal with stressful situations, and ultimately, reduce the high rates of internalizing disorders affecting teenagers in the U.S. Future studies should work to evaluate the impact of Reset Outdoors sessions by measuring changes in health outcomes among student participants. Quantitative evidence of the effectiveness of the programs on student outcomes would complement the qualitative results in the current study. Both qualitative and quantitative evidence of the effectiveness of the program could provide a research base to (1) refine future iterations of the Reset Outdoors intervention at VTHS, (2) support the dissemination of similar interventions at other schools and facilities, and (3) advocate for more resources to expose young people to nature-based self-care programs.

## 5. Conclusions

With the rise in mental health concerns among children and youth in the United States, schools are increasingly giving attention to services and programs that promote mental and emotional well-being among students. This study examined the experiences and perceptions of teachers who participated in one such program situated at the intersection of self-care and nature-based interventions. The pilot program of focus in this study took place at a vocational technical high school and involved eight teachers participating in the Reset Outdoors organization’s Intro to Well-Being program. While including a relatively small cohort of participants, this study, nonetheless, highlighted the benefits observed by teachers, especially for students’ self-awareness, emotional expression, stress, and connection with peers and teachers. In particular, teachers highlighted the positive benefits of the outdoor silent walk, a nature-based mindfulness activity that anchored the Intro to Well-Being sessions. Additionally, teachers observed improved student engagement and a more positive classroom climate after the Well-Being sessions, benefits which transcended the individual mental and emotional health of students, and supported broader academic goals more generally. While teachers had recommendations for improving program effectiveness in future incarnations, they were uniformly positive in their assessment of the program, underscoring the potential for implementation of such a program in other school contexts.

## Data Availability

The data presented in this study are available on request from the corresponding author, conditional on IRB requirements.

## Abbreviations

The following abbreviations are used in this manuscript:

VTHS	Vocational Technical High School
SEL	Social–Emotional Learning
